# Supplementary material for: Structure of SARS-CoV-2 M protein in lipid nanodiscs
Source: eLife. 2022 Oct 20;11:e81702. doi: 10.7554/eLife.81702 (PMC9642992; doi:10.7554/eLife.81702)
Supplement: Supplementary file 1. [file elife-81702-supp1.docx]

| **Data collection** | **SARS-CoV-2 M** |
| --- | --- |
| PDB | 8CTK |
| EMDB | 26993 |
| Total movies | 7588 |
| Magnification | 165,000 x |
| Voltage (KV) | 300 |
| Electron exposure (e^-^/Å^2^) | 50 |
| Defocus range (um) | -0.5 to -1.2 |
| Super resolution pixel size (Å^2^) | 0.3635 |
| Binned pixel size (Å^2^) | 0.727 |
| **Processing** |  |
| Initial particle images (no.) | 2,007,561 |
| Final particle images (no.) | 64,966 |
| Map resolution Masked (Å, FSC = 0.143) | 3.52 |
| Symmetry imposed | C2 |
| **Refinement** |  |
| Model resolution (Å, FSC = 0.143 / FSC = 0.5) | 3.2 /3.5 |
| Map-sharpening B factor (Å^2^) | -145 |
| Composition |  |
| Number of atoms | 3042 |
| Number of protein residues | 376 |
| Number of ligands | 0 |
| RMS deviations |  |
| Bond lengths (Å) | 0.003 |
| Bond angles (Å) | 0.690 |
| Validation |  |
| MolProbity score | 1.47 |
| Clashscore | 4.37 |
| Ramachandran plot |  |
| Favored (%) | 96.24 |
| Allowed (%) | 3.76 |
| Disallowed (%) | 0 |
| Rotamer outliers (%) | 0 |
| Mean B factor (Å^2^) |  |
| Protein | 42.42 |
